# Reference gene selection for real-time qPCR in European flounder (*Platichthys flesus*) using organ-specific RNA-seq data

**DOI:** 10.1007/s11033-024-10105-7

**Published:** 2024-11-21

**Authors:** Konrad Pomianowski, Artur Burzyński

**Affiliations:** Department of Genetics and Marine Biotechnology, Institute of Oceanology, Polish Academy of Sciences, Powstańców Warszawy 55, Sopot, 81-712 Poland

**Keywords:** HKG, Housekeeping genes, Differential expression analysis, Fish

## Abstract

**Background:**

The European flounder is readily chosen as an experimental subject and model in physiological and ecotoxicological studies mostly because of its adaptability to laboratory conditions. Many studies utilise a quantitative PCR (qPCR) approach to ascertain the expression of target genes under experimental conditions. Such an approach relies heavily on the selection of reference genes with stable expression. Yet certain housekeeping genes are commonly used in this role, often without due consideration of their overall expression patterns. Therefore, new approaches should be developed to identify stable reference genes for a given species and to expand the general pool of genes suitable for the reference in qPCR analysis.

**Methods and results:**

Here RNA-seq data of nine flounder organs led to identify four candidate genes of the most stable expression. It was achieved by differential expression analysis and *tritoconstrictor* script. Specific primers were designed for the complete ORF as well as for qPCR analysis. RT-qPCR efficiencies were tested on ORF amplicon templates. Most of the genes tested showed good amplification in a wide range of template dilutions (10^7^-10^1^), with a correlation coefficient (R^2^) ranging from 0.991 to 0.998 and a consistent efficiency (E) (Sybr Green I staining and TaqMan molecular probe).

**Conclusions:**

The proposed approach based on differential expression analysis and a new bioinformatic tool is an appropriate selection method of candidates for reference genes in qPCR. The proposed approach, combining differential expression analysis with a new bioinformatics tool, provides an effective method for selecting reference gene candidates for qPCR. As a result, we can propose four genes (*polr2f*, *yif1a*, *sf3b6*, *uba52*), each with a set of validated primers, as suitable for consideration as reference genes in qPCR analysis in European flounder, an emerging model species.

## Introduction

Despite the increasing availability of RNA-seq technology, real-time reverse transcription polymerase chain reaction (RT-qPCR) remains a commonly used method in molecular biology research to determine gene expression. This technique is particularly useful for studying changes in the expression of selected genes under varying experimental conditions. In RT-qPCR, amplification products can be detected either: i) by its intercalation with a fluorescent dye (i.e. Sybr Green I) or much more specifically, by ii) molecular probes, i.e. TaqMan, working on the principle of probe hybridization (fluorescent labeled oligonucleotide) to the complementary sequence of the gene of interest and its release after amplification [1]. In both cases, fluorescent signals are emitted and detected on every PCR cycle, and the point above which the increase in fluorescence is exponential is called the quantification Cycle (Cq). There are different normalization strategies for RT-qPCR data [2], but one of the most commonly used is normalization to the reference gene, which is also utilized in the $\:{2}^{-\varDelta\:\varDelta\:Ct}$ method [3], for determining relative fold gene expression of samples based on Cq values of target and reference genes. The reference gene should meet specific criteria, including stable expression levels that are not affected by experimental conditions and low variability between organs and physiological states of the organism [4]. The reference genes most commonly used are housekeeping genes (HKG), such as encoding β-actin, glyceraldehyde-3-phosphate dehydrogenase (*GAPDH*) and 18S ribosomal RNA. Historically, the same genes were also used as references in northern blots or end-point RT-qPCR assays [2]. Over the years, the expression of “classic” references proved to be affected by experimental conditions, and using them in particular cases may lead to incorrect results [5]. Therefore, it is essential to identify new stable candidates for reference genes that would show only minimal susceptibility to experimental conditions.

The European flounder is a species distributed in European coastal waters from the White Sea in the north to the Mediterranean and the Black Sea in the south. This flatfish is frequently used as an experimental subject because the species is easily adaptable to laboratory conditions. It is chosen as a model in research aiming investigation of physiological processes in fish and in stress response studies [6, 7], as well as in ecotoxicological research [8–10]. Recent studies on wild flounders from polluted areas suggest that this species could also be used to track cancer defense mechanisms [11]. Various housekeeping genes were commonly used as internal standards when investigating gene expression through the RT-qPCR in flounder, including β-actin, 18S, α-tubulin, elongation factor 1 [8, 10], the F-actin capping protein β subunit and ubiquitin [8, 12]. However, information about their expression is available for a limited number of flounder organs only.

One popular way to choose reference genes is by utilizing different algorithms implemented in the geNorm, BestKeeper, and NormFinder software packages [13–15]. This approach requires input data (Cq values, gene expression data) obtained during the amplification of genes that are suspected to have a stable expression in the particular organ and under a particular condition. However, it involves outlays of laboratory work and chemical reagents and allows testing of a limited number of candidate reference genes. In this study, we propose a different approach, based on the analysis of available RNA-seq data across nine European flounder’s organs [16], to identify the genes of the most stable expression. For this purpose, bioinformatic analysis was applied for the differential expression across organs.

## Materials and methods

### RNA-seq analysis

The results of RNA-seq obtained from the eyeball, brain, intestine, spleen, heart, liver, head kidney, gonads, and skin of the flounder described previously [16] were used. Total RNA from reported organs was extracted as described in details in Pomianowski et al. [16, 17]. The assembly was performed in Trinity assembler version 2.9.1 [18] with default parameters. To quantify the transcripts, we used the following scripts distributed with Trinity. First, the align_and_estimate_abundance.pl perl script was used utilizing Salmon software [19]. Then, the resulting quant.sf files generated by Salmon were subject to normalization procedures [20], as implemented in the abundance_estimates_to_matrix.pl perl script. The gene level statistics were obtained by adding the counts for all isoforms [21]. The exon boundaries for the candidate transcripts were inferred by aligning the selected transcripts to the reference genome of Japanese flounder (*Paralichthys olivaceus*) as in [16]. Finally, the table generated by the tritoconstrictor* script [16] was used to identify the genes with stable expression across all the organs (Table 1). The following criteria were taken into account. First, the variability of expression across the sampled organs was evaluated by calculating the standard deviation (SD) of the TPM (Transcript Per Million) value of each of the transcripts in each organ. Then, the measure of variability was normalized by dividing it by the average TPM value for all organs in each given gene. This relative SD value was then used to sort the table. Top 26 genes were examined in detail, taking into account the reliability of their annotation, the integrity of their transcript assembly, and the level of expression. The genes with the average expression below 20 TPM were not considered. The final choice was also informed by the inferred role and the structure of the transcript. The preference was given to putative reference genes, with the intron-exon structure allowing efficient primer design, allowing the target to be located close to the end of the transcript, with at least one primer located at the exon boundary. The final choice constituted three genes: *polr2f*,* yif1a*, *sf3b6* (Table 1). In addition to them a gene encoding ubiquitin, *uba52*, frequently used as a reference in transcriptomic studies, was also identified in the table and subject to the same primer design procedure.

**Table 1:** Expression of selected transcripts in European flounder organs. Twenty-six transcripts showing relatively small variability across organs are shown. Transcripts selected as candidates for reference genes were underlined. The expression is presented in TPM units. The table is sorted by increasing relative standard deviation (relSD). Comparative values for the *uba52* gene, a typical reference gene, are shown in the last row

Product name	G gonad	jel intestine	Mozg brain	Ngl head kidney	oko eyeball	se heart	skd skin bottom part	skg skin upper part	sled spleen	W liver	avg	SD	relSD
Putative myotubularin-related protein 9-like	35	26	26	24	28	27	26	35	29	19	28	5	0.17
DNA-directed RNA polymerases I, II, and III subunit RPABC2	75	56	55	49	71	76	69	52	75	48	63	11	0.18
Serine/threonine-protein phosphatase	130	128	98	95	76	77	112	93	87	112	101	19	0.19
Putative charged multivesicular body protein 3	22	27	30	18	25	23	29	27	18	18	24	5	0.20
Protein YIF1A isoform X1 n 2, simple transcript	35	24	29	21	30	23	33	24	19	26	26	5	0.20
Serine and arginine rich splicing factor 2b	27	18	16	25	27	24	27	25	17	20	23	4	0.20
MORC family CW-type zinc finger protein 2	24	27	18	20	19	15	14	23	24	18	20	4	0.21
Protein sodium potassium root defective 2	29	32	25	24	28	30	15	31	32	37	28	6	0.21
Transmembrane EMP24 domain-containing protein 9	61	81	65	58	54	80	54	62	48	94	66	14	0.22
Polyadenylate-binding protein	73	56	38	59	54	80	57	82	52	65	62	14	0.22
Protein YIPF	16	35	26	22	24	17	22	27	26	24	24	5	0.22
Zinc finger Ran-binding domain-containing protein 2	23	21	39	36	36	31	30	23	31	23	29	7	0.22
Diablo-like protein 2	45	40	62	42	36	50	52	27	37	50	44	10	0.22
heterogeneous nuclear ribonucleoprotein K isoform X3	49	62	43	38	65	57	28	63	57	58	52	12	0.23
vacuolar protein sorting-associated protein 45 isoform X2	14	26	20	23	18	13	22	21	30	22	21	5	0.24
Polymerase (RNA) II (DNA directed) polypeptide E	47	34	43	33	30	39	58	28	51	39	40	10	0.24
DDRGK domain-containing protein 1	36	29	21	21	30	31	32	17	27	37	28	7	0.24
splicing factor, suppressor of white-apricot homolog	22	35	18	39	31	41	37	25	36	29	31	8	0.25
Mitotic spindle-associated MMXD complex subunit MIP18	73	51	53	36	52	47	50	30	46	66	51	13	0.25
Transcription factor BTF3 family member	109	104	140	96	91	137	101	73	100	168	112	28	0.25
Sorting nexin-3	25	45	31	43	27	27	43	26	41	27	34	8	0.25
synaptosomal-associated protein 29	19	20	27	22	16	12	29	17	23	18	20	5	0.26
Vacuolar protein-sorting-associated protein 25	28	31	25	34	30	24	40	35	30	13	29	7	0.26
RNA polymerase I and III subunit D	37	29	19	23	16	29	28	21	26	17	24	6	0.26
RNA binding motif protein 39b	26	18	15	30	27	25	23	27	34	16	24	6	0.26
Splicing factor 3B subunit 6	82	70	134	94	94	67	71	63	99	68	84	22	0.26
Ubiquitin A-52 residue ribosomal protein fusion product 1	1085	2094	1259	1815	1005	2410	3128	2183	3184	5353	2352	1304	0.55

avg – average; SD – standard deviation; relSD – sd divided by average TPM value

* https://github.com/aburzynski/tritoconstrictor

### Primer and molecular probes design

**Fig. 1:**
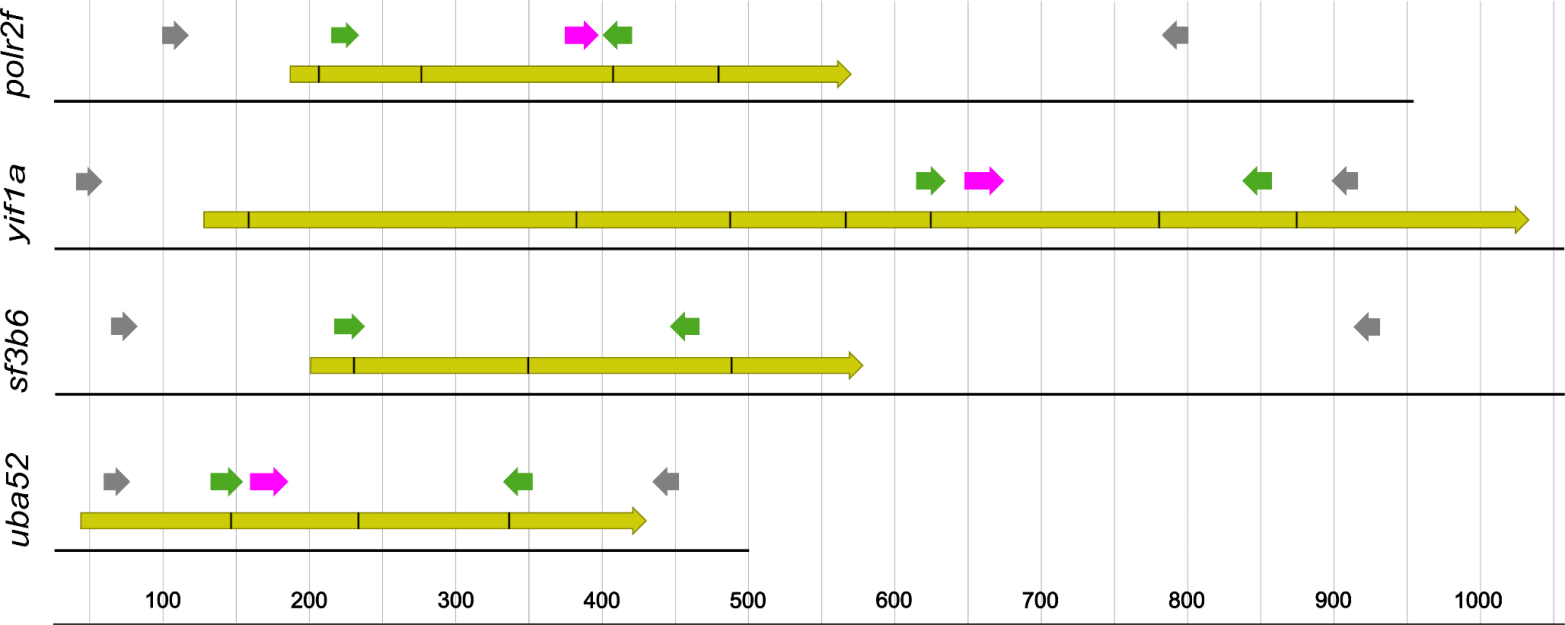
Schematic representation of gene intron-exon structure of candidates for reference genes: *polr2f*, *yif1a*, *sf3b6* and *uba52*. Marked exon-exon junctions were established in comparison with genomic sequences of Japanese flounder (*Paralichthys olivaceus*). The ORF primer (grey), reference gene primer (green) and molecular probe (pink) location sites are marked above the sequence

Primers were designed to amplify four candidate genes: *polr2f*, *yif1a*, *sf3b6* and *uba52*. These primers allow the detection of the RT-qPCR products using the DNA intercalating dye. Additionally, molecular TaqMan probes [22] were also designed for three of the genes (*polr2f*, *yif1a* and *uba52*), hybridizing to the target transcript between the previously designed primers (Fig. 1). This approach enables alternative detection method with TaqMan probes, thus enhancing experimental design possibilities, such as multiplexing qPCR. Primers and probes (Table 2) were designed using Primer3 software v. 2.4.0 [23, 24], with default input settings. At least one primer was located at an exon-exon junction, preventing gDNA amplification. Furthermore, additional primer pairs were designed to amplify a fragment of each transcript templates encompassing the qPCR target, by reverse transcription PCR (rtPCR) (Table 2; Fig. 1). These amplicons were subsequently used as templates to find the optimal conditions for qPCR.

**Table 2:** Primers and probe sequences, RT-qPCR parameters, standard curve range

Gene name and Gene Bank accession number	Product name	Primer and probe sequence (5’→3’)		Product size (bp)	Correlation coefficient (*R*^2^)	Melt curve peak (°C)	Standard curve range
*polr2f* NC_084960.1	DNA-directed RNA polymerases I-III subunit RPABC2	Target sequence	F: ATGGAGACTTCGATGACGCC	206	0.997	88.0	10^7^-10^1^
			R: TGGAGCACACATCGCAATCT				
			P: CCGAGTGCTGGGGACACGGGCGC		-	-	-
		ORF amplicon template	F: CTCATGTGGGTTCGTGTT	701	-	-	-
			R: CCTGGTGGTCGTTAGTCA				
*yif1a* NC_084952.1	protein YIF1A isoform X1	Target sequence	F: TGCAGCAAAGGTTCAGTCCA	243	0.998	86.7	10^7^-10^1^
			R: CACATGAGGACCAGGCAAGG				
			P: TGTGTGCCAGCACTGCCCTCGTGTGGA		-	-	-
		ORF amplicon template	F: GAAGAAGAAAGAAGGCGT	876	-	-	-
			R: GTGGGAGATAGAGGGAAG				
*sf3b6* NC_084962.1	Splicing factor 3B subunit 6	Target sequence	F: CGAAACGCGCTAATATCCGATT	250	0.991	82.3	10^5^-10^2^
			R: ACCACCAAGTAACGGTTGCA				
		ORF amplicon template	F: GGCATAGTCGAAAACAGG	867	-	-	-
			R: TTTAGGTCACATCGCAGG				
*uba52* NC_084953.1	Ubiquitin A-52 residue ribosomal protein fusion	Target sequence	F: CCAGGACAAGGAAGGAATTCCC	220	0.994	86.1	10^7^-10^1^
			R: CAGACGGGCATAGCATTTGC				
			P: VIC-TCAGCAGCGTCTGATCTTCGCCGGCA-MGB		0.987	-	10^7^-10^4^
		ORF amplicon template	F: AGACGTTGACGGGGAAGA	393	-	-	-
			R: AGAGCCAATTCAGCCAAA				

### RT-qPCR conditions

When planning and carrying out the optimization process, we followed the MIQE guidelines [25], for providing all relevant conditions and assay characteristics. Having PCR templates brings certain advantages for the determination of optimal parameters for RT-qPCR. It saves the initial RNA template and protects it from repeated thawing, which could otherwise lead to degradation, ultimately affecting the results. The PCR template obtained after cDNA amplification is more resistant to handling (thawing) and it is possible to precisely determine its concentration based on the length and spectrophotometric concentration. The cDNA synthesis and templates PCR were carried out in the Eppendorf Mastercycler X50S (Eppendorf, Hamburg, Germany). For cDNA synthesis 370 ng of total RNA extracted from spleen was reverse transcribed using the SensiFAST^tm^ cDNA Synthesis Kit (Bioline, London, UK) and diluted 1:10. The PCR was then performed in a total volume of 50 µl in Q5 reaction buffer (New England Biolabs, Ipswich, USA) containing 1.0 U/µl of Q5 High-Fidelity DNA polymerase, 200 µM of dNTP mix, 2.5 mM of each primer and 11.5 µl of cDNA solution. The PCR amplification temperatures were tested in gradient ranging from 55 °C to 68 °C for each ORF primer pair. The final PCR program consisted of 30 s denaturation at 98 °C, followed by 40 cycles at 98 °C (10 s), amplification at 65.2 °C (*sf3b6*, *polr2f*, *uba52*) or 62.4 °C (*yif1a*) for 30 s, elongation at 72 °C (30 s: *polr2f*, *uba52*, *yif1a*; 25 s: *sf3b6*) and final elongation of 2 min. (72 °C). After optimization, a specific product was obtained for each target, as confirmed by 1.5% agarose gel electrophoresis. The products were purified using Extractme DNA Clean-up Kit (Blirt, Gdańsk, Poland) and their concentration was estimated spectrophotometrically (Epoch™ Microplate Spectrophotometer, BioTek, Winooski, USA).

**Fig. 2:**
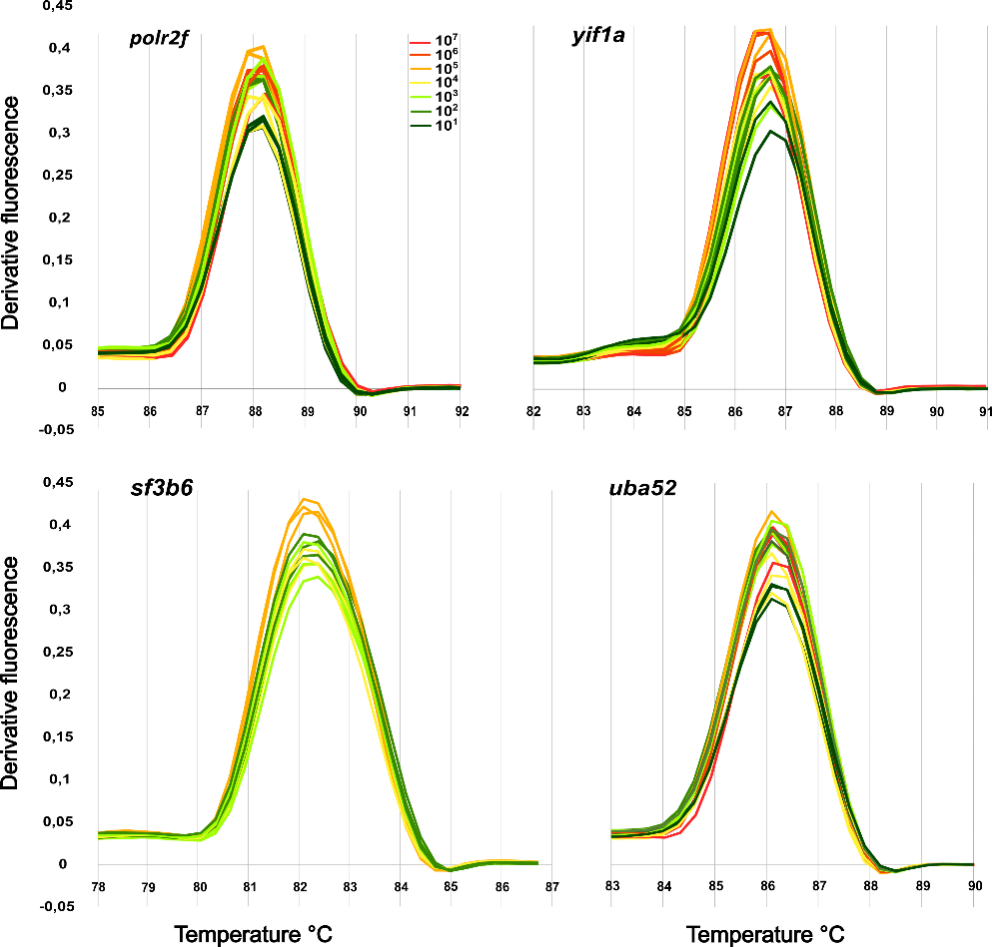
Melting curves of the qPCR product (Sybr Green I dye labeling) of candidates for European flounder reference genes. Curves were made at every dilution point of the standard curve where amplification occurred: *polf2r*, *yif1a*, *uba52* (10^7^-10^1^) and *sf3b6* (10^5^-10^2^)

The real-time PCR efficiency for pairs of qPCR primers for each reference genes candidate was estimated from standard curves made from serial, ten-fold dilutions of each template (10^8^-10^1^), starting at the absolute concentration of 1254 × 10^8^ (*polr2f*), 312 × 10^8^ (*yif1a*), 128 × 10^8^ (*sf3b6*), and 2054 × 10^8^ (*uba52*) molecules/µl. The reactions were performed in the Eco Real-Time PCR System (Illumina San Diego, CA, USA) using the SensiFAST^tm^ SYBR No-ROX kit (Bioline, London, UK). Each qPCR was run in triplicate in the total volume of 10 µl SensiFAST mix containing 0.4 mM (*polr2f*, *yif1a*,* sf3b6*) or 0.2 mM (*uba52*) of each primer and the template at variable concentration. The PCR program started with polymerase activation (3 min, 95 °C) followed by 40 cycles at 95 °C (5 s), 65–66 °C of annealing temp. (10 s) and final extension taking 5 s (72 °C). The specificity of the reaction was confirmed by the melting curve (94 °C, 60 °C, 94 °C) with each step lasting 15 s (Fig. 2). Additionally, one target, located in the *uba52* gene, was tested in reactions containing a TaqMan MGB-NFQ molecular probe with VIC as fluorescent reporter dye (Thermo Scientific, Waltham, MA, USA) and the same pair of primers as in tested by Sybr Green I labeling (Table 2). qPCRs were run in a total volume of 10 µL ABsolute QPCR Mix (Thermo Scientific, Waltham, MA, USA), 0.3 µM of each primer, 0.25 µM of each molecular probe and template starting at the absolute concentration of approximately 10^11^ molecules/µl. The following protocol was used: 95 °C for 15 min, followed by 40 PCR cycles of 15 s at 95 °C (denaturation), 30 s at 65 °C (primer annealing) and 30 s at 72 °C (elongation).

### Targets optimization and tests

To validate the assays by checking the stability and repeatability of Cq values qPCR efficiency and expression stability were evaluated using total RNA extracts from different organs. RNA derived from the eyeball and skin of nine individuals was used, including the one that provided the sequencing results. Total RNA was reverse transcribed as described in the previous paragraph, and serial ten-fold dilutions of the obtained cDNA (10^7^-10^4^) were used in qPCR.

## Results and discussion

Typical qPCR studies rely on a limited set of candidate genes, assumed to show stable expression [13–15]. Extending this set should be beneficial, increasing the reliability of these studied. Identifying genes stably expressed across different organs does not guarantee their suitability as references, but since different organs ultimately represent different physiological states, such RNA-Seq data can extend the pool of potential reference genes without much experimental work.

Differential expression analysis of RNA-Seq data [18, 19] (Table 1), allowed identification of three well-defined, stably expressed genes across nine flounder organs. These genes correspond to different housekeeping functions, including transcription (*polr2f)*, cellular transport (*yif1a*), and post-transcriptional processes (*sf3b6).* The established target (*uba52*) is known not only for signaling via ubiquitination, but it also encodes ribosomal protein so it is involved in translation. The three selected genes were never used as reference genes previously in that species [8, 9, 12].

**Fig. 3:**
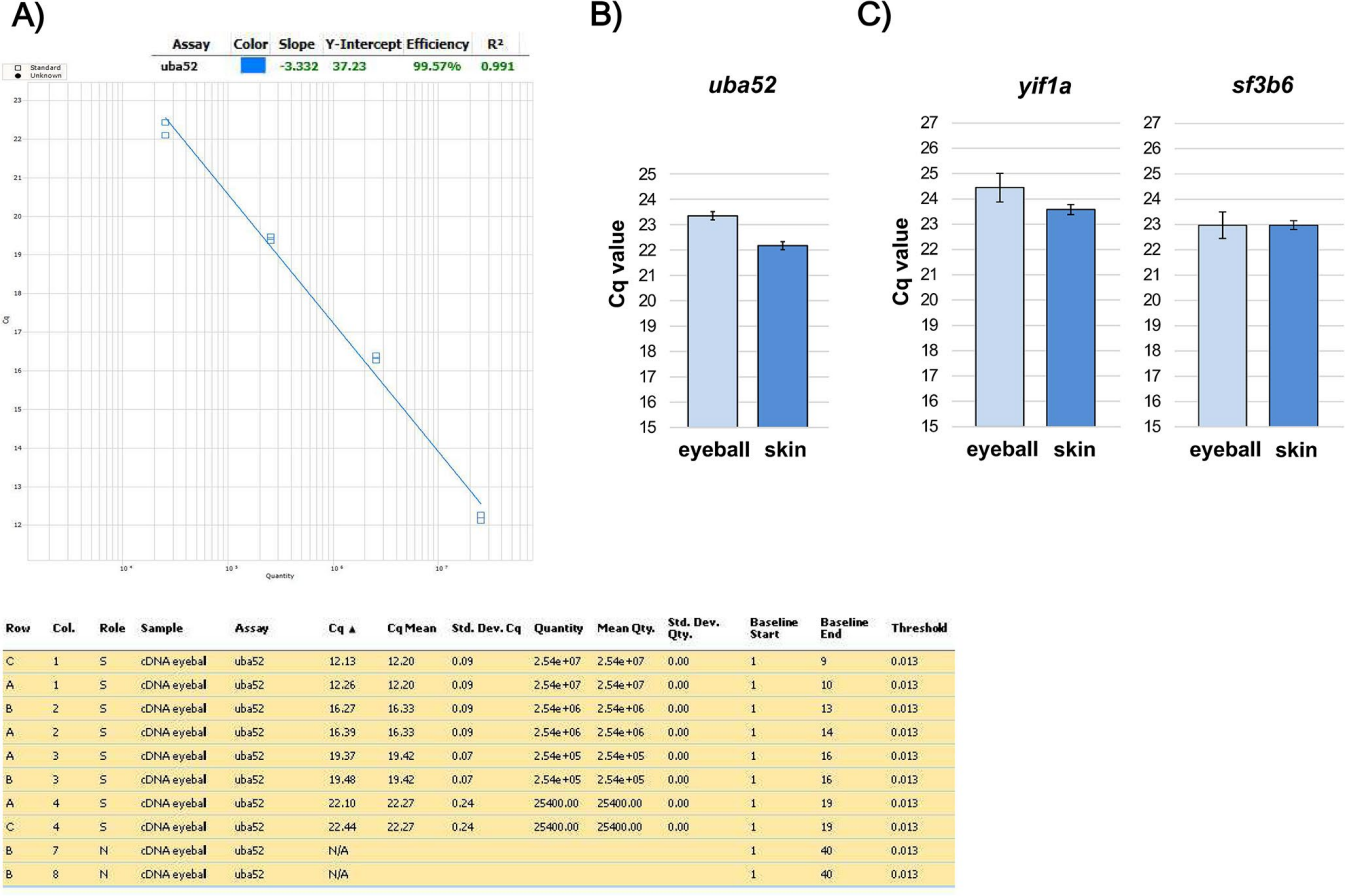
**A**) The efficiency of qPCR using a TaqMan molecular probe designed for the *uba52* target, calculated from a standard curve (10⁷-10⁴) generated by serial ten-fold dilutions of cDNA (reverse-transcribed RNA extracted from *P. flesus* eyeballs). **B**) Cq values obtained from flounder eyeball (*n* = 4) and skin (*n* = 2) samples using qPCR with a TaqMan molecular probe for the *uba52* target. **C**) Cq values obtained from flounder eyeball (*n* = 2) and skin (*n* = 2) samples using qPCR with SYBR Green labeling for both *yif1a* and *sf3b6* molecular targets. Values are presented as ± SEM

Implementation of a qPCR assay should involve finding the optimal conditions and assessing the stability of expression. To this end, after the specific primers were designed for all genes, optimal results were obtained at annealing temperatures of 65–66 °C, achieving reaction efficiencies (E) of 105-127%. Three targets (*polr2f*, *yif1a*, *uba52*) were amplifiable across seven orders of magnitude (10^7^ to 10^1^, R² 0.994–0.998), while *sf3b6* was reliably amplified over only four orders of magnitude (10^5^ to 10^2^, R² 0.991). Specificity was confirmed by the single melting point of the qPCR product for each target (Fig. 2). Moreover, TaqMan probe tests for *uba52* showed an efficiency of 102% and R² values of 0.987 for the 10^7^ to 10^4^ dilution range. Efficiency tests on cDNA obtained from total RNA confirmed good qPCR efficiency for *uba52* (efficiency: 99.57%; R² 0.991, Fig. 3). Stability tests (Cq values) for *uba52*, *yif1a*, and *sf3b6* showed satisfactory results in selected organs (Fig. 3).

Using cDNA amplicon templates for qPCR efficiency tests, we were able to check a wide range of template concentrations in multiple replicates, with limited RNA usage. Even with simple Sybr Green I labeling, which is prone to primer-dimer artefacts, the assays showed high amplification and correct R² values, with a specific, narrow melting points of qPCR products for each dilution and each target. Higher than expected efficiency in three out of four investigated genes may be a side effect of using purified templates, but it should not affect the assays run on RNA.

TaqMan probe tests for *uba52* (Fig. 3A, B) confirmed the experimental feasibility of using this more robust (but also more expensive) reporting system. However, since probe labeling and eventual multiplexing is hardware-specific, more testing is needed for the new reference candidate genes, should anybody wish to apply them in their qPCR assays involving European flounder, an emerging model species.

## Data Availability

No datasets were generated or analysed during the current study.
